# Sociodemographic and Anthropometric Determinants of Body Dissatisfaction in a Sample of Spanish Adolescents: The EHDLA Study

**DOI:** 10.3390/ejihpe16100146

**Published:** 2026-09-23

**Authors:** Diana Ramos-Camino, Camila Miño, José Adrián Montenegro-Espinosa, Fiorella Quiroz-Cárdenas, Lucille Ridgell, Rodrigo Yáñez-Sepúlveda, Jorge Olivares-Arancibia, Jacqueline Páez-Herrera, Dong Keon Yon, José Francisco López-Gil

**Affiliations:** 1 School of Medicine, Universidad Espíritu Santo, Samborondón 092301, Ecuador; dianaramos@uees.edu.ec; 2 Universidad Internacional para el Desarrollo (UNINDE), 06080 Badajoz, Spain; camilamino501@gmail.com; 3 Universidad Autónoma del Paraguay, Asunción 111215, Paraguay; josemontenegro733@gmail.com; 4 Vicerrectoría de Investigación y Postgrado, Universidad de Los Lagos, Osorno 5290000, Chile; fiorella.quiroz@yahoo.com; 5 Department of Health Research, Icen Cognis, 38370 La Matanza de Acentejo, Spain; 6 Carver University, Orlando, FL 32804, USA; lucilleridgell@gmail.com; 7 Faculty of Education and Social Sciences, Universidad Andres Bello, Viña del Mar 2520000, Chile; rodrigo.yanez.s@unab.cl; 8 AFySE Group, Research in Physical Activity and School Health, School of Physical Education, Faculty of Education, Universidad de las Américas, Santiago 8320000, Chile; 9 Grupo Investigación Efidac, Escuela Educación Física, Pontificia Universidad Católica de Valparaíso, Valparaíso 2370200, Chile; jacqueline.paez@pucv.cl; 10 Center for Digital Health, Medical Science Research Institute, Kyung Hee University College of Medicine, Seoul 02447, Republic of Korea; 11 Department of Precision Medicine, Kyung Hee University College of Medicine, Seoul 02447, Republic of Korea; 12 Department of Pediatrics, Kyung Hee University Medical Center, Kyung Hee University College of Medicine, Seoul 02447, Republic of Korea; 13 Faculty of Health Sciences, Universidad Tecnológica Atlántico Mediterráneo—UTAMED, 29590 Málaga, Spain

**Keywords:** body mass index, sex differences, maternal education, overweight, psychosocial factors, Spain

## Abstract

Body dissatisfaction is prevalent among adolescents and linked to adverse outcomes. This study examined sociodemographic and anthropometric factors associated with body dissatisfaction in Spanish adolescents. Cross-sectional data from the Eating Healthy and Daily Life Activities (EHDLA) study (N = 1378 students aged 12–17 years) were analyzed. Body dissatisfaction was assessed using the Body Image Dimensional Assessment (BIDA) instrument (overall index and three subscales). Variables included age, sex, socioeconomic status, immigrant status, family structure, parental education, area of residence, and body mass index. Robust multivariable linear models adjusted for all a priori covariates were fitted. Multivariable complete-case models included *n* = 747 of 1378 adolescents. Younger age, female sex, and excess weight were associated with higher body dissatisfaction on one or more BIDA dimensions. Excess weight showed the largest standardized associations and was linked to all BIDA dimensions (*p* < 0.001). Maternal educational attainment was not significant in the fully adjusted primary models but remained associated with lower Body Dissatisfaction Score in a reduced-model sensitivity analysis. No significant associations were observed for socioeconomic status, immigrant status, family structure, or area of residence in the primary models. Younger adolescents, female adolescents, and those with excess weight represent subgroups at greater risk of body dissatisfaction. These findings underscore the need for targeted prevention and intervention strategies addressing both anthropometric and sociodemographic risk factors during adolescence.

## 1. Introduction

Adolescence is a transitional stage between childhood and adulthood, characterized by profound physical, psychological, biological, and social changes (Banati et al., 2025). These changes may be particularly challenging, as adolescents undergo processes of identity formation and self-esteem development (Harter, 2012). Large-scale international survey data indicate that between 55% and 65% of adolescents report some degree of body dissatisfaction, with female adolescents consistently exhibiting higher rates than male adolescents across all countries studied (Napp, 2026). Distress related to body dissatisfaction affects not only how adolescents perceive themselves but may also have adverse consequences for both mental and physical health (Hock et al., 2025).

Body dissatisfaction is defined as an individual’s dissatisfaction with their own physical appearance or overall body shape (Stice & Shaw, 2002). In adolescents, it has been assessed using a range of instruments. One of the most widely used figure rating scales is the Stunkard Figure Rating Scale, which presents silhouettes ordered from the smallest body to the largest body and asks respondents to select the figures representing their perceived and ideal body shapes; the discrepancy between the two is interpreted as an indicator of body dissatisfaction (Stunkard et al., 1983; Scagliusi et al., 2006). However, silhouette-based measures may be relatively coarse and insufficiently sensitive for adolescent populations (Segura-García et al., 2012). For this reason, more recent instruments such as the Body Image Dimensional Assessment (BIDA) have been used in adolescent research (Segura-García et al., 2012; Sánchez-Miguel et al., 2021). The BIDA uses four figurative representations to assess adolescents’ current body perception, personal body ideal, perceived sexual attractiveness, and the body shape perceived as typical among same-sex peers. From these selections, three body dissatisfaction dimensions and a global index can be calculated (Sánchez-Miguel et al., 2021; Segura-García et al., 2012). Its psychometric properties have been validated specifically in Spanish adolescents (Sánchez-Miguel et al., 2021). Therefore, its potential contribution lies in distinguishing personal, attractiveness-related, and peer-referenced body evaluations. However, whether this multidimensional structure provides incremental information beyond established figure rating scales requires direct empirical comparison.

Body dissatisfaction has increased markedly in recent years. In a study of Spanish adolescents, Lopez-Muley et al. (2024) reported a substantial rise between 2016 and 2021, from 44.2% to 60.4%, indicating growing dissatisfaction with body image among adolescents. In that study, body dissatisfaction was assessed using the Contour Drawing Rating Scale, a silhouette-based instrument in which participants select the figure that best represents their current body and the one corresponding to their desired body; the discrepancy between both figures is used as an indicator of body dissatisfaction (Lopez-Muley et al., 2024). Similarly, a cross-national study including adolescents from 24 countries found that male adolescents reported higher levels of body dissatisfaction in countries such as Italy (39.9%), the United States (37.7%) and Greece (35.2%), whereas higher prevalence among female adolescents was observed in the Czech Republic (61.8%), Slovenia (56.8%), and Italy (55.2%) (Al Sabbah et al., 2009). Body dissatisfaction in adolescents is not determined by a single factor but by multiple external and individual influences, including external factors such as media exposure and comments from family members or peers, and individual factors such as age, sex, nationality, body mass index (BMI), and socioeconomic characteristics, among others (Jiménez Boraita et al., 2021; Robbins et al., 2017). Contemporary body image research conceptualizes body dissatisfaction not only as an individual response to demographic or anthropometric characteristics, but also as a phenomenon shaped by interpersonal and sociocultural processes. Family and peer feedback, appearance-related comments, social comparison, the internalization of culturally valued body ideals, and exposure to appearance-focused digital media may influence how adolescents evaluate their bodies. Social media may be particularly relevant because it provides frequent opportunities for appearance comparison and exposure to idealized or digitally modified images (Fardouly & Vartanian, 2016; Nerini et al., 2024). Similarly, family members, peers, and romantic partners may reinforce or challenge appearance standards through body-related comments and interpersonal validation (Fornaini et al., 2021; Rodgers et al., 2014). From this perspective, sociodemographic and anthropometric characteristics may be associated with body dissatisfaction partly through broader psychological and social processes rather than operating as isolated determinants.

Evidence indicates that it varies according to sociodemographic characteristics, particularly age, with some studies reporting higher levels among older adolescents (Chen et al., 2010; Pelegrini et al., 2014). Beyond sociodemographic characteristics, anthropometric factors such as BMI have also been consistently associated with body dissatisfaction during adolescence, with higher BMI linked to greater dissatisfaction with body image (Fernández-Bustos et al., 2019).

Sex differences in body dissatisfaction have also been consistently reported, although patterns vary across populations. Female adolescents tend to aspire to a thin body shape, whereas male adolescents more often endorse a larger and more muscular physique as the ideal (Dion et al., 2015). Accordingly, female adolescents more frequently report a desire to lose weight and higher levels of body dissatisfaction (Latiff et al., 2018), as well as a greater body distortion (Jung & Jun, 2022). However, this pattern is not universal, as higher levels of body dissatisfaction among male adolescents have been reported in Sri Lankan adolescents (Liyanage et al., 2021), driven by the desire to appear bigger and more robust.

Given that adolescence represents a critical developmental period marked by profound physical, psychological, and social changes, body dissatisfaction may have negative implications for adolescent health and psychosocial well-being (Neumark-Sztainer et al., 2006). Although previous research has identified several correlates of body dissatisfaction (Chen et al., 2010; Pelegrini et al., 2014), important gaps remain in understanding how sociodemographic and anthropometric factors are distributed across different dimensions of body dissatisfaction. Because the BIDA distinguishes dissatisfaction relative to adolescents’ personal body ideal, perceived sexual attractiveness, and same-sex peer norms, examining these dimensions separately may help characterize whether correlates are similarly observed across these reference frameworks (Sánchez-Miguel et al., 2021; Segura-García et al., 2012). In this context, the aim of the present study was to examine the sociodemographic and anthropometric correlates of body dissatisfaction in a sample of Spanish adolescents, considering both the global BIDA index and its three dimension-specific scores.

## 2. Materials and Methods

### 2.1. Study Design and Population

This study is a cross-sectional secondary analysis based on data from the Eating Healthy and Daily Life Activities (EHDLA) Study. The EHDLA Study is a research project designed to collect information on overweight and its potential correlates, including sociodemographic, environmental, lifestyle, health, cognitive, and psychological factors among adolescents aged 12–17 years. The study population comprised secondary school students from three educational institutions in the Valle de Ricote (Region of Murcia, Spain). The detailed methodology of the EHDLA Study has been described elsewhere (López-Gil, 2022). The initial sample comprised 1378 adolescents, of whom 49.3% were male. The full sample was used to describe participant characteristics and variable-specific missingness. Complete BIDA data were available for 855 participants (62.0%), whereas 523 participants (38.0%) had missing data on one or more BIDA scores. After excluding participants with missing data on any covariate included in the models, 747 adolescents (54.2% of the initial sample) remained for the complete-case multivariable analyses.

Data were collected during physical education classes throughout the 2021–2022 academic year. Participation required written informed consent from parents or legal guardians of the selected adolescents. Both families and participants received detailed information on the study objectives, planned assessments, and questionnaires to be administered, and adolescents provided assent to participate. The study was conducted in accordance with the principles of the Declaration of Helsinki. The research protocol was approved by the Bioethics Committee of the University of Murcia (ID 2218/2018, approved on 18 February 2019) and by the Ethics Committee of Albacete University Hospital Complex and the Albacete Integrated Care Management (ID 2021–86, approved on 23 November 2021).

### 2.2. Instruments

#### 2.2.1. Sociodemographic Factors

All sociodemographic variables available in the EHDLA database were entered simultaneously into the analysis to allow a comprehensive assessment and minimize the risk of omitted-variable bias. Age was calculated from self-reported date of birth and categorized into two groups: 12–14 years and 15–17 years. Sex was self-reported using the response options male and female; gender identity was not assessed. Race/ethnicity was self-reported and originally coded into six categories (Caucasian, Latin, Arab, Gypsy, Afro-American, and Asian). Because of small numbers of participants in non-Caucasian categories, these were collapsed into a binary variable (Caucasian vs. Non-Caucasian) for multivariable analyses, with Caucasian as the reference group. Immigrant status was defined as meeting at least one of the following criteria: (a) being born outside Spain, (b) having at least one parent born in another country, or (c) having at least one parent of foreign origin. Socioeconomic status (SES) was assessed using the Family Affluence Scale (FAS-III), a six-item instrument including: (a) family car ownership (0 = no, 1 = yes, one, 2 = yes, two or more); (b) having one’s own bedroom (0 = no, 1 = yes); (c) number of computers at home (0 = none, 1 = one, 2 = two, 3 = more than two); (d) number of bathrooms in the household (0 = none, 1 = one, 2 = two, 3 = more than two); (e) dishwasher ownership (0 = no, 1 = yes); and (f) number of family holidays abroad during the previous year (0 = none, 1 = once, 2 = twice, 3 = more than twice). Total scores ranged from 0 to 13 and were classified as low (0–2), medium (3–5), or high (≥6) socioeconomic status.

The EHDLA study also collected information on parental or legal guardian educational attainment, categorized as: (a) completed primary education, (b) incomplete primary education, (c) completed secondary education, (d) incomplete secondary education, (e) incomplete higher education, and (f) completed higher education. Information was also available on type of school, classified as public or publicly funded private (concertado). Both school types operate under state regulation; however, publicly funded private schools are privately managed and partially financed with public resources. Parental education and school type were analyzed as separate covariates and were not incorporated into the composite socioeconomic status measure. Adolescents also reported the number of people living in their household and family structure, originally categorized as: (a) two-parent family, (b) reconstituted family, (c) same-sex parent family, (d) single-father household, (e) single-mother household, (f) extended family, and (g) adoptive family. For analysis, these categories were recoded into four groups: (a) nuclear, (b) single-parent, (c) extended, and (d) diverse family structure. For multivariable models, family structure was further dichotomized as nuclear versus non-nuclear (single-parent, extended, or diverse). Area of residence was classified as urban (>5000 inhabitants) or rural (≤5000 inhabitants) (Ministerio de Medio Ambiente y Medio Rural y Marino & Unidad de Análisis y Prospectiva, 2009).

#### 2.2.2. Anthropometric Factors

Adolescents’ BMI was calculated from weight and height measurements collected in the EHDLA study using standardized procedures, by dividing body weight (kg) by height squared (m^2^). BMI values were converted into age- and sex-specific z-scores and used to classify weight status according to the 2007 World Health Organization (WHO) growth reference for school-aged children (De Onis, 2007). For multivariable analyses, BMI status was dichotomized as excess weight (overweight or obesity) versus no excess weight (underweight or normal weight).

#### 2.2.3. Body Dissatisfaction

Body dissatisfaction was assessed using the BIDA, previously validated in Spanish adolescents (Sánchez-Miguel et al., 2021). The instrument consists of four items that assess subjective body image perception through the selection of figurative representations along a continuous scale ranging from 1.8 (smallest body representation) to 5.2 (largest body representation). The four BIDA items were denoted as Q1, representing the adolescent’s current body; Q2, the personally ideal body; Q3, the body perceived as most attractive to the opposite sex; and Q4, the body perceived as typical among same-sex and same-age peers. Because the figurative scale ranges from 1.8 to 5.2, its total range of 3.4 was used as the denominator to standardize the discrepancy scores. The Body Dissatisfaction Score (BD) was calculated as:

$$BD=\frac{\left(Q1-Q2\right)\times 100}{3.4}$$

The Sexual Body Dissatisfaction Score (SxBD) was calculated as:

$$SxBD=\frac{\left(Q1-Q3\right)\times 100}{3.4}$$

The Comparative Body Dissatisfaction Score (CBD) was calculated as:

$$CBD=\frac{\left(Q1-Q4\right)\times 100}{3.4}$$

Each discrepancy score ranged from −100% to 100%. Positive values indicated that the current body estimate was larger than the corresponding reference figure, whereas negative values indicated that it was smaller. The global BIDA index was calculated as the mean of the absolute values of the three discrepancy scores:

$$BIDA\;index=\frac{\left|BD\right|+\left|SxBD\right|+\left|CBD\right|}{3}$$

The global index ranged from 0 to 100, with higher values indicating greater overall body dissatisfaction. For example, if Q1 = 4.2, Q2 = 3.5, Q3 = 3.8, and Q4 = 4.0, the resulting scores would be BD = 20.59, SxBD = 11.76, and CBD = 5.88, producing a global BIDA index of 12.74. In the present study, the global BIDA index was considered the primary outcome variable, with the three subscales analyzed separately as secondary outcomes to explore dimension-specific associations with sociodemographic and anthropometric factors.

### 2.3. Statistical Analysis

Categorical variables were summarized as frequencies (n) and percentages (%). Because residual diagnostics indicated non-normality of the BIDA outcomes, primary multivariable associations were estimated using robust linear regression (MM-estimation; robustbase::lmrob, KS2014 setting) with all a priori sociodemographic and anthropometric covariates entered simultaneously. Ordinary least-squares (Gaussian) models with the same full covariate set, and robust models after backward elimination (α = 0.20), were examined as sensitivity analyses. For each model we report unstandardized coefficients (B) with 95% confidence intervals (CIs) and *p*-values, together with standardized betas (β) to facilitate comparison of relative association magnitudes. 

Multivariable analyses were conducted using a complete-case approach (listwise deletion) and were restricted to participants with complete data for the BIDA outcomes and all covariates included in the models (*n* = 747). Participants with missing data on any model variable were excluded from the inferential analyses. Although this approach may reduce the analytical sample and introduce potential selection bias, it ensured that all estimates were obtained from the same set of participants. To evaluate potential selection bias, sociodemographic and anthropometric characteristics of participants included in the complete-case analyses (*n* = 747) were compared with those of excluded participants (Table S4). All statistical analyses were conducted using R statistical software (version 4.3.2; R Core Team, Vienna, Austria) with RStudio (version 2023.12.1+402; Posit, Boston, MA, USA). A two-sided *p*-value < 0.05 was considered statistically significant in all analyses.

## 3. Results

Table 1 presents the descriptive characteristics of the adolescents included in the study. Of the 1378 adolescents included in the full sample, 49.3% were male and 50.7% were female, with 54.5% aged 12–14 years and 45.5% aged 15–17 years. Compared with excluded adolescents, those retained in the complete-case multivariable sample (*n* = 747) were more often aged 12–14 years (65.1% vs. 42.0%), female (54.5% vs. 46.1%), and, among participants with available family-structure data, living in nuclear families (84.5% vs. 74.5%). They also differed in number of siblings and household size (Table S4; all *p* < 0.05). Among participants with available data, the prevalence of excess weight (25.6% vs. 26.7%; *p* = 0.714) and SES distribution (*p* = 0.943) did not differ significantly between included and excluded participants. In the full sample (Table 1), the majority were of Caucasian ethnicity (83.2%), native-born (75.2%), and resided in urban areas (74.1%). Most adolescents came from nuclear family structures (81.2%) and attended public schools (81.5%). With respect to socioeconomic status, 52.7% were classified in the medium category, 25.5% in the high category, and 21.9% in the low category. Regarding weight status, 66.5% of participants had normal weight, 17.0% were classified as overweight, 9.0% were classified in the “obesity” category, and 7.5% were classified as underweight. The median Body Dissatisfaction Score was 5.9 (interquartile range [IQR]: 0.0 to 17.6), indicating that the typical participant perceived their current body as somewhat heavier than their personal ideal. Both the Sexual Body Dissatisfaction Score and the Comparative Body Dissatisfaction Score had a median of 0.0, although their interquartile ranges differed (IQR: −5.9 to 14.7 and −8.8 to 14.7, respectively), suggesting considerable variability in body image perceptions related to attractiveness and peer comparison. The median BIDA index was 10.8 (IQR: 5.9 to 19.6). Complete BIDA data were available for 855 participants, whereas 523 participants (38.0%) had missing data on one or more BIDA scores. After additionally excluding participants with missing data on one or more covariates included in the models, 747 adolescents were included in the complete-case multivariable analyses.

Table 2 shows the multivariable associations between sociodemographic and anthropometric factors and the BIDA index from the primary fully adjusted robust models. No statistically significant associations were observed between the BIDA index and the sociodemographic variables examined. In contrast, adolescents with excess weight had significantly higher BIDA index values than those without excess weight (*B* = 6.93; 95% CI: 5.42 to 8.44; *p* < 0.001; β = 0.61).

Table S1 presents the multivariable associations with the Body Dissatisfaction Score from the primary fully adjusted robust models. Significant associations were observed for age, sex, and BMI status. Adolescents aged 15–17 years reported lower body dissatisfaction than those aged 12–14 years (*B* = −2.30; 95% CI: −4.41 to −0.19; *p* = 0.033; β = −0.13). Female adolescents showed higher levels of body dissatisfaction than male adolescents (*B* = 2.38; 95% CI: 0.39 to 4.37; *p* = 0.020; β = 0.14). Maternal university education was not statistically significant in the fully adjusted primary model (*B* = −1.94; 95% CI: −4.39 to 0.50; *p* = 0.120; β = −0.11), although it remained significant in the backward-selection sensitivity model (*B* = −2.50; 95% CI: −4.67 to −0.33; *p* = 0.024). BMI status was the strongest predictor: adolescents with excess weight had higher scores than those without excess weight (*B* = 13.69; 95% CI: 11.42 to 15.96; *p* < 0.001; β = 0.79).

Table S2 presents the multivariable associations with the Sexual Body Dissatisfaction Score from the primary fully adjusted robust models. Adolescents aged 15–17 years had lower scores than those aged 12–14 years (*B* = −2.47; 95% CI: −4.83 to −0.10; *p* = 0.041; β = −0.14). Other sociodemographic covariates were not statistically significant. Adolescents with excess weight showed substantially higher scores than those without excess weight (*B* = 16.24; 95% CI: 13.69 to 18.79; *p* < 0.001; β = 0.92).

Table S3 presents the multivariable associations with the Comparative Body Dissatisfaction Score from the primary fully adjusted robust models. No sociodemographic covariate reached statistical significance. Adolescents with excess weight showed substantially higher scores than those without excess weight (*B* = 17.53; 95% CI: 14.80 to 20.26; *p* < 0.001; β = 0.87).

## 4. Discussion

The present study examined the sociodemographic and anthropometric correlates of body dissatisfaction in a sample of Spanish adolescents using the multidimensional structure of the BIDA. The central contribution of the findings was not the replication of the well-established associations between body dissatisfaction, female sex, and excess weight, but the characterization of how these associations were distributed across different dimensions of body dissatisfaction. Younger age and female sex were associated with higher dissatisfaction relative to adolescents’ personal body ideal in the fully adjusted primary models. These associations did not reach statistical significance for dissatisfaction referenced to perceived sexual attractiveness or same-sex peers, with the exception of a younger-age association for the sexual attractiveness dimension. In contrast, excess weight was the only factor consistently associated with the global BIDA index and all three dimension-specific outcomes. These findings suggest that the correlates of body dissatisfaction may not be uniformly observed across the different reference frameworks adolescents use when evaluating their bodies. The median BIDA index was 10.8 points within a scale ranging from 0 to 100, suggesting that, overall, the sample did not exhibit markedly elevated levels of body dissatisfaction, although considerable variability was observed across individuals and subgroups. Because appearance-related social comparison, internalization of appearance ideals, body-related comments, weight stigma, media exposure, and family communication were not directly assessed, the mechanisms underlying the observed associations cannot be determined from the present data. Therefore, the explanations discussed below should be interpreted as plausible hypotheses rather than demonstrated pathways. The dimension-specific pattern of associations may be understood in relation to the distinct reference frameworks captured by the BIDA. The Body Dissatisfaction Score reflects adolescents’ personal body ideal, whereas the Sexual and Comparative Body Dissatisfaction Scores refer to perceived attractiveness and same-sex peer norms, respectively (Sánchez-Miguel et al., 2021; Segura-García et al., 2012). In the fully adjusted primary models, maternal educational attainment was not significantly associated with any BIDA outcome, although a protective association with the personal-ideal dimension was observed in a reduced-model sensitivity analysis; any family-level interpretations (e.g., maternal modeling) therefore remain tentative and were not directly assessed (Handford et al., 2018). In contrast, the association between excess weight and all BIDA outcomes suggests that weight status may be relevant across personal, attractiveness-related, and peer-referenced evaluations. Self-discrepancy and sociocultural perspectives provide plausible frameworks involving ideal internalization and appearance comparison (Higgins, 1987; Rodgers et al., 2014; Thompson et al., 1999). The different pattern observed for the Sexual Body Dissatisfaction Score may also reflect perceived attractiveness and interpersonal feedback, although evidence from adult relationships may not be directly applicable to adolescents (Fornaini et al., 2021). Therefore, these interpretations should be considered tentative.

The age-related pattern observed for the personal-ideal dimension contrasts with studies reporting higher body dissatisfaction among older adolescents, including Taiwanese and Ethiopian samples (Chen et al., 2010; Jebero et al., 2025). These discrepancies may reflect meaningful differences in age range, cultural context, and assessment instruments across studies. In particular, the use of the BIDA instrument, which captures multiple dimensions of body image perception simultaneously, may yield different patterns compared with unidimensional silhouette-based tools such as the Contour Drawing Rating Scale, and comparisons should therefore be interpreted with caution.

Wang et al. (2019) reported relatively stable longitudinal trajectories of body dissatisfaction across adolescence, with certain subgroups maintaining elevated but non-escalating levels over time. Although the cross-sectional design of the present study does not allow conclusions about within-person changes, the lower Body Dissatisfaction Scores observed among older adolescents are compatible with the possibility that body dissatisfaction does not necessarily increase progressively throughout adolescence. Several explanations may account for the lower Body Dissatisfaction Scores observed among older adolescents. Pubertal changes may increase attention to physical appearance and perceived discrepancies between adolescents’ bodies and socially internalized ideals, particularly during early adolescence (Tiggemann, 2005). However, pubertal timing was not assessed in the present study, and this explanation therefore remains tentative. Differences between age groups may also reflect cohort effects or age-related changes in appearance-based social comparison and self-concept (Lacroix et al., 2022; Mitchell et al., 2021). Nevertheless, these processes were not directly measured, and the cross-sectional design does not allow us to determine whether the observed differences represent developmental changes over time. The age association reached statistical significance for the Body Dissatisfaction Score and the Sexual Body Dissatisfaction Score, but not for the global BIDA index or the Comparative Body Dissatisfaction Score. 

The sex-specific pattern observed for the personal-ideal dimension is consistent with previous evidence reporting greater body image concerns among female adolescents, including findings from Jung and Jun (2022), who reported greater body image distortion among female adolescents in a South Korean sample, and with Tiggemann (2005), who documented elevated body shape dissatisfaction among Australian female adolescents. In the Brazilian context, Gonzaga et al. (2023) attributed higher body dissatisfaction in female adolescents to intensified sociocultural pressure to attain a thinner body shape, a pattern that appears to transcend national contexts. In contrast, Liyanage et al. (2021) reported higher body dissatisfaction among male adolescents in Sri Lanka, a finding attributed to male adolescents’ desire to appear larger and more robust, highlighting that the direction of sex differences in body dissatisfaction may be context-dependent and shaped by culturally specific body ideals. Because female and male adolescents tend to internalize body ideals differently (with female adolescents more frequently oriented toward thinness and male adolescents toward muscularity and physical size), these differences may reflect broader cultural pressures that encourage female adolescents from early ages to place greater emphasis on physical appearance (Carvalho et al., 2020). In the present study, female sex was significantly associated with the Body Dissatisfaction Score, whereas the associations with the sexual and comparative body dissatisfaction dimensions did not reach statistical significance. Therefore, in this sample, the statistically significant sex difference was observed only for dissatisfaction relative to adolescents’ personal body ideal.

Although maternal educational attainment was not significant in the fully adjusted primary models, a protective association with the Body Dissatisfaction Score in sensitivity analyses would be consistent with previous studies linking lower maternal education to greater body dissatisfaction (Kapoor et al., 2022; Rashmi et al., 2016). Several mechanisms may potentially explain this association, including differences in health literacy, family communication, parental attitudes toward appearance, parenting practices, access to health-related resources, and maternal modeling of body image and weight-related behaviors. Higher maternal educational attainment may be associated with greater access to health-related information and more supportive guidance regarding body image and weight management (Kapoor et al., 2022). Previous research has also identified maternal modeling as one possible influence on body image concerns during adolescence (Handford et al., 2018). These processes were not directly assessed in the present study and should therefore be considered alternative hypotheses rather than established mechanisms underlying the observed association. In sensitivity analyses, a maternal-education association was retained only for the Body Dissatisfaction Score; it was not significant for SxBD or CBD in either the primary or the reduced models. Therefore, this dimension-specific finding should be interpreted cautiously.

The consistent association between excess weight and all BIDA outcomes aligns with evidence from Spanish and Taiwanese adolescents linking higher BMI to greater body dissatisfaction and a stronger desire for a thinner body (Chen et al., 2008; Fernández-Bustos et al., 2019). In Spanish adolescent populations, both the BIDA instrument and the Stunkard Figure Rating Scale have consistently shown that BMI is associated with greater body dissatisfaction during adolescence (Jiménez Boraita et al., 2021; Sánchez-Miguel et al., 2021). Several psychological and sociocultural processes may potentially contribute to the association between excess weight and body dissatisfaction. According to self-discrepancy and sociocultural perspectives, appearance-related social comparison and the internalization of thin or athletic body ideals may increase the perceived discrepancy between adolescents’ bodies and culturally valued appearance standards (Higgins, 1987; Thompson et al., 1999). Weight stigma, body-related comments, peer interactions, and media exposure may also contribute to this association. However, these processes were not directly assessed in the present study and should therefore be considered plausible hypotheses rather than established explanations for the observed association.

Several limitations should be considered when interpreting the findings of the present study. First, the use of self-administered questionnaires may have introduced social desirability and recall bias, particularly for items related to body image perception. Second, the sample was drawn exclusively from three educational institutions in the Valle de Ricote region of Murcia, which limits generalizability to other adolescent populations, both within Spain and internationally. Third, nearly half of the initial sample was excluded from multivariable analyses because of missing BIDA and/or covariate data (analytic *n* = 747/1378; 54.2%). Comparison of included versus excluded participants (Table S4) showed differences in age, sex, family structure, number of siblings, and household size, indicating possible selection bias for these sociodemographic estimates; however, the prevalence of excess weight and the SES distribution did not differ significantly between groups. Fourth, the cross-sectional design precludes any inference regarding causality or directionality of the observed associations. Fifth, the study did not assess several psychological and sociocultural variables relevant to adolescent body dissatisfaction, including appearance-related teasing, internalization of appearance ideals, body surveillance, appearance-based social comparison, self-esteem, and social media exposure (Fardouly & Vartanian, 2016; Menzel et al., 2010; Rodgers et al., 2014; Slater & Tiggemann, 2010). Because these variables were not measured, their potential roles as confounders or mediators of the observed associations could not be examined. Consequently, the reported associations should be understood as representing only part of a broader explanatory framework of body image development during adolescence. Sixth, pubertal timing (a well-established correlate of body image concerns in this age group) was not assessed and therefore could not be controlled for in the analyses. Seventh, immigrant status was operationalized as a composite variable conflating first- and second-generation immigration, which are conceptually distinct constructs that may relate differently to body dissatisfaction. Eighth, sex was recorded using binary male and female response options, whereas gender identity was not assessed. Therefore, the findings should not be interpreted as reflecting gender identity and may not be generalizable to gender-diverse adolescents. Finally, because no other body image measure was included, we could not compare the BIDA scores with those obtained from other established instruments or determine whether the BIDA provided additional information beyond these measures.

The present study also has several strengths that must be mentioned. An important strength of the present study was the use of the multidimensional BIDA, previously validated in Spanish adolescents, which allowed dissatisfaction relative to personal body ideals, perceived sexual attractiveness, and same-sex peer norms to be examined separately. Although the BIDA has previously been applied in adolescent samples, mainly in studies focused on behavioral and nutritional variables such as physical activity and diet (Gualdi-Russo et al., 2022; Menghi et al., 2025), the present study extended its application by characterizing sociodemographic and anthropometric patterns across its different dimensions. This multidimensional scoring structure enabled the characterization of distinct patterns of association across personal and socially referenced dimensions of body dissatisfaction. The dimension-specific patterns observed in the present study indicate that the three BIDA dimensions were not fully interchangeable, as some correlates were limited to the personal-ideal dimension, whereas excess weight was associated across all dimensions. This finding illustrates the conceptual usefulness of distinguishing personal and socially referenced body evaluations.

The relatively large sample size, the comprehensive assessment of sociodemographic characteristics, and the use of objectively measured anthropometric data according to standardized WHO criteria further strengthen the study. Additionally, the simultaneous inclusion of all sociodemographic predictors in the multivariable model minimized the risk of omitted-variable bias and allowed the independent contribution of each factor to be assessed while controlling for the influence of the others.

## 5. Conclusions

In the present study, younger age, female sex, and higher BMI status (excess weight) were associated with higher levels of body dissatisfaction across one or more BIDA dimensions in fully adjusted robust models. Maternal educational attainment was not significant in the primary full models, although a protective association with the Body Dissatisfaction Score emerged in a reduced-model sensitivity analysis. Socioeconomic status, immigrant status, family structure, area of residence, and race/ethnicity were not significantly associated with any body dissatisfaction outcome. Among all factors examined, BMI status emerged as the strongest and most consistent predictor, with adolescents with excess weight showing substantially higher dissatisfaction across all dimensions, including personal ideal, sexual attractiveness, and peer-referenced body comparisons. Taken together, these findings indicate that sociodemographic correlates were concentrated mainly in dissatisfaction relative to adolescents’ personal body ideal, whereas excess weight was consistently associated with both personal and socially referenced dimensions. This pattern suggests that body dissatisfaction may not be adequately understood as a uniform construct, as its correlates may vary according to the reference framework adolescents use when evaluating their bodies.

These findings highlight the importance of developing more specific school-based prevention strategies targeting body dissatisfaction, particularly during early adolescence. Potential components could include activities aimed at reducing appearance-related teasing and weight stigma, strengthening media literacy, decreasing appearance-based social comparison and the internalization of unrealistic body ideals, and promoting positive body image, body appreciation, and respect for body diversity rather than focusing exclusively on weight management. Family-level components could also encourage supportive communication and reduce appearance- and weight-related comments in the home environment. Because these psychosocial processes were not directly assessed, these recommendations should be considered potential directions for prevention rather than interventions directly supported by the present findings. Future research should examine the directionality of the associations identified using longitudinal designs and explore additional social and contextual factors that may influence body dissatisfaction.

## Figures and Tables

**Table 1:** Descriptive data of the study participants.

Variable	N = 1378
Age group	
12–14 years old	751 (54.5%)
15–17 years old	627 (45.5%)
Sex	
Male	680 (49.3%)
Female	698 (50.7%)
SES	
Low	243 (21.9%)
Medium	585 (52.7%)
High	283 (25.5%)
Missing	267
Number of siblings	
None	122 (11.0%)
One	585 (52.7%)
Two or more	404 (36.4%)
Missing	267
Immigrant status	
Native	836 (75.2%)
Immigrant	275 (24.8%)
Missing	267
Number of people at home	
Two or less	186 (16.7%)
Three	782 (70.4%)
Four or more	143 (12.9%)
Missing	267
Race/ethnicity	
Caucasian	924 (83.2%)
Non-Caucasian	187 (16.8%)
Missing	267
Type of schooling	
Public	1123 (81.5%)
Private with public funds	255 (18.5%)
Parental education (mother)	
Non-university education	800 (73.2%)
University education	293 (26.8%)
Missing	285
Parental education (father)	
Non-university education	828 (78.4%)
University education	228 (21.6%)
Missing	322
Type of family	
Nuclear	902 (81.2%)
Single-parent	73 (6.6%)
Extended	37 (3.3%)
Diverse	99 (8.9%)
Missing	267
Area of residence	
Urban	823 (74.1%)
Rural	288 (25.9%)
Missing	267
BMI status	
Underweight	95 (7.5%)
Normal weight	838 (66.5%)
Overweight	214 (17.0%)
“Obesity”	114 (9.0%)
Missing	117
Body dissatisfaction (score)	5.9 (0.0, 17.6)
Missing	523
Sexual body dissatisfaction (score)	0.0 (−5.9, 14.7)
Missing	523
Comparative body dissatisfaction (score)	0.0 (−8.8, 14.7)
Missing	523
BIDA index (score)	10.8 (5.9, 19.6)
Missing	523

Data expressed as median (interquartile range) or n (%). “Obesity” is written with quotation marks because it reflects the clinical BMI category and is referred as neutral, consistent with current weight-inclusive. BIDA, Body Image Dimensional Assessment; EHDLA, Eating Healthy and Daily Life Activities; BMI, body mass index; SES, socioeconomic status.

**Table 2:** Sociodemographic and anthropometric correlates of the BIDA index (EHDLA study, *n* = 747).

Predictor	*B* (95% CI)	β	*p*-Value
Age group			
12–14 years old	Reference		
15–17 years old	−1.32 (−2.72, 0.07)	−0.12	0.063
Sex			
Male	Reference		
Female	−0.03 (−1.34, 1.28)	0.00	0.959
SES			
Low	Reference		
Medium	0.68 (−1.10, 2.46)	0.06	0.455
High	0.07 (−2.03, 2.16)	0.01	0.951
Number of siblings			
None	Reference		
One	0.22 (−2.52, 2.96)	0.02	0.875
Two or more	1.02 (−1.86, 3.90)	0.09	0.487
Immigrant status			
Native	Reference		
Immigrant	0.14 (−1.96, 2.24)	0.01	0.895
Number of people at home			
Two or less	Reference		
Three	1.44 (−0.92, 3.81)	0.13	0.231
Four or more	0.88 (−2.14, 3.91)	0.08	0.567
Race/ethnicity			
Caucasian	Reference		
Non-Caucasian	−1.39 (−3.82, 1.05)	−0.12	0.264
Type of schooling			
Public	Reference		
Private with public funds	−1.16 (−2.84, 0.51)	−0.10	0.174
Parental education (mother)			
Non-university education	Reference		
University education	−0.58 (−2.20, 1.03)	−0.05	0.478
Parental education (father)			
Non-university education	Reference		
University education	−1.26 (−2.98, 0.45)	−0.11	0.149
Type of family			
Nuclear	Reference		
Non-nuclear	−0.26 (−2.17, 1.66)	−0.02	0.794
Area of residence			
Urban	Reference		
Rural	−0.87 (−2.37, 0.62)	−0.08	0.252
BMI status			
No excess weight	Reference		
Excess weight	6.93 (5.42, 8.44)	0.61	<0.001

Data are unstandardized beta coefficients (*B*) with 95% confidence intervals (CIs), standardized betas (β), and *p*-values from multivariable robust linear models (MM-estimation; lmrob, KS2014 setting) including all a priori covariates simultaneously (complete-case analytic sample, *n* = 747). *p*-values are reported to three decimal places; values <0.001 are shown as <0.001. BMI, body mass index; SES, socioeconomic status.

## Data Availability

The data presented in this study are available on request from the corresponding author due to their sensitive nature.

## Supplementary Materials

The following supporting information can be downloaded at: https://www.mdpi.com/article/10.3390/ejihpe16100146/s1. Table S1: Sociodemographic and anthropometric correlates of the Body Dissatisfaction Score; Table S2: Sociodemographic and anthropometric correlates of the Sexual Body Dissatisfaction Score; Table S3: Sociodemographic and anthropometric correlates of the Comparative Body Dissatisfaction Score; Table S4: Comparison of participants included in the complete-case multivariable analyses (*n* = 747) versus those excluded due to missing data on BIDA outcomes and/or covariates (EHDLA study).
