# Supplementary material for: Sociodemographic and Anthropometric Determinants of Body Dissatisfaction in a Sample of Spanish Adolescents: The EHDLA Study
Source: Eur J Investig Health Psychol Educ. 2026 Sep 23;16(10):146. doi: 10.3390/ejihpe16100146 (PMC13703428; doi:10.3390/ejihpe16100146)
Supplement: Supplementary file 1 [file ejihpe-16-00146-s001.zip › ejihpe-4373236-supplementary.pdf]

Sociodemographic and Anthropometric Determinants of Body Dissatisfaction in a Sample of Spanish Adolescents: The EHDLA Study

Supplementary Materials

Contents

Table S1. Sociodemographic and anthropometric correlates of the Body Dissatisfaction Score (primary fully adjusted robust linear models).
Table S2. Sociodemographic and anthropometric correlates of the Sexual Body Dissatisfaction Score (primary fully adjusted robust linear models).
Table S3. Sociodemographic and anthropometric correlates of the Comparative Body Dissatisfaction Score (primary fully adjusted robust linear models).
Table S4. Comparison of participants included versus excluded from complete-case multivariable analyses.

Table S1. Sociodemographic and anthropometric correlates of the Body Dissatisfaction Score (EHDLA study, n = 747).

Predictor	B (95% CI)	β	p
**Age group**			
12–14 years old	Reference	—	—
15–17 years old	-2.30 (-4.41, -0.19)	-0.13	0.033
**Sex**			
Male	Reference	—	—
Female	2.38 (0.39, 4.37)	0.14	0.019
**SES**			
Low	Reference	—	—
Medium	0.22 (-2.48, 2.93)	0.01	0.871
High	0.50 (-2.67, 3.67)	0.03	0.756
**Number of siblings**			
None	Reference	—	—
One	1.41 (-2.75, 5.58)	0.08	0.507
Two or more	1.28 (-3.09, 5.65)	0.07	0.566
**Immigrant status**			
Native	Reference	—	—
Immigrant	2.30 (-0.88, 5.48)	0.13	0.157
**Number of people at home**			
Two or less	Reference	—	—
Three	0.54 (-3.03, 4.12)	0.03	0.766
Four or more	-2.58 (-7.17, 2.01)	-0.15	0.270
**Race/ethnicity**			
Caucasian	Reference	—	—
Non-Caucasian	-0.90 (-4.55, 2.75)	-0.05	0.630
**Type of schooling**			
Public	Reference	—	—
Private with public funds	-0.25 (-2.81, 2.30)	-0.01	0.847
**Parental education (mother)**			
Non-university education	Reference	—	—
University education	-1.94 (-4.39, 0.50)	-0.11	0.120
**Parental education (father)**			
Non-university education	Reference	—	—
University education	-1.19 (-3.80, 1.42)	-0.07	0.370
**Type of family**			
Nuclear	Reference	—	—
Non-nuclear	-0.23 (-3.13, 2.67)	-0.01	0.877
**Area of residence**			
Urban	Reference	—	—
Rural	0.30 (-1.97, 2.57)	0.02	0.794
**BMI status**			
No excess weight	Reference	—	—
Excess weight	13.69 (11.42, 15.96)	0.79	<0.001

Data are unstandardized beta coefficients (B) with 95% confidence intervals (CI), standardized betas (β), and p-values from multivariable robust linear models (MM-estimation; lmrob, KS2014 setting) including all a priori covariates simultaneously (complete-case analytic sample, n = 747). p-values are reported to three decimal places; values <0.001 are shown as <0.001. BMI, body mass index; SES, socioeconomic status.

Table S2. Sociodemographic and anthropometric correlates of the Sexual Body Dissatisfaction Score (EHDLA study, n = 747).

Predictor	B (95% CI)	β	p
**Age group**			
12–14 years old	Reference	—	—
15–17 years old	-2.47 (-4.83, -0.10)	-0.14	0.041
**Sex**			
Male	Reference	—	—
Female	-1.96 (-4.19, 0.26)	-0.11	0.084
**SES**			
Low	Reference	—	—
Medium	-0.93 (-3.94, 2.09)	-0.05	0.547
High	0.37 (-3.17, 3.90)	0.02	0.839
**Number of siblings**			
None	Reference	—	—
One	3.29 (-1.36, 7.94)	0.19	0.166
Two or more	3.09 (-1.79, 7.97)	0.18	0.215
**Immigrant status**			
Native	Reference	—	—
Immigrant	2.52 (-1.01, 6.05)	0.14	0.162
**Number of people at home**			
Two or less	Reference	—	—
Three	-0.60 (-4.60, 3.39)	-0.03	0.767
Four or more	-4.95 (-10.08, 0.19)	-0.28	0.059
**Race/ethnicity**			
Caucasian	Reference	—	—
Non-Caucasian	-1.25 (-5.32, 2.83)	-0.07	0.549
**Type of schooling**			
Public	Reference	—	—
Private with public funds	-0.82 (-3.68, 2.04)	-0.05	0.574
**Parental education (mother)**			
Non-university education	Reference	—	—
University education	-1.44 (-4.19, 1.31)	-0.08	0.304
**Parental education (father)**			
Non-university education	Reference	—	—
University education	-1.93 (-4.85, 0.99)	-0.11	0.196
**Type of family**			
Nuclear	Reference	—	—
Non-nuclear	-2.77 (-6.03, 0.49)	-0.16	0.096
**Area of residence**			
Urban	Reference	—	—
Rural	0.84 (-1.70, 3.37)	0.05	0.519
**BMI status**			
No excess weight	Reference	—	—
Excess weight	16.24 (13.69, 18.79)	0.92	<0.001

Data are unstandardized beta coefficients (B) with 95% confidence intervals (CI), standardized betas (β), and p-values from multivariable robust linear models (MM-estimation; lmrob, KS2014 setting) including all a priori covariates simultaneously (complete-case analytic sample, n = 747). p-values are reported to three decimal places; values <0.001 are shown as <0.001. BMI, body mass index; SES, socioeconomic status.

Table S3. Sociodemographic and anthropometric correlates of the Comparative Body Dissatisfaction Score (EHDLA study, n = 747).

Predictor	B (95% CI)	β	p
**Age group**			
12–14 years old	Reference	—	—
15–17 years old	-1.17 (-3.71, 1.38)	-0.06	0.369
**Sex**			
Male	Reference	—	—
Female	2.09 (-0.30, 4.49)	0.10	0.086
**SES**			
Low	Reference	—	—
Medium	-0.54 (-3.76, 2.68)	-0.03	0.742
High	-0.18 (-3.97, 3.61)	-0.01	0.926
**Number of siblings**			
None	Reference	—	—
One	1.37 (-3.59, 6.33)	0.07	0.588
Two or more	2.46 (-2.76, 7.68)	0.12	0.356
**Immigrant status**			
Native	Reference	—	—
Immigrant	2.86 (-0.96, 6.69)	0.14	0.143
**Number of people at home**			
Two or less	Reference	—	—
Three	2.38 (-1.88, 6.64)	0.12	0.274
Four or more	-1.62 (-7.11, 3.87)	-0.08	0.563
**Race/ethnicity**			
Caucasian	Reference	—	—
Non-Caucasian	-2.14 (-6.57, 2.29)	-0.11	0.344
**Type of schooling**			
Public	Reference	—	—
Private with public funds	-1.26 (-4.34, 1.81)	-0.06	0.421
**Parental education (mother)**			
Non-university education	Reference	—	—
University education	0.07 (-2.88, 3.02)	0.00	0.962
**Parental education (father)**			
Non-university education	Reference	—	—
University education	-1.98 (-5.12, 1.16)	-0.10	0.216
**Type of family**			
Nuclear	Reference	—	—
Non-nuclear	-1.63 (-5.12, 1.85)	-0.08	0.358
**Area of residence**			
Urban	Reference	—	—
Rural	-0.15 (-2.87, 2.57)	-0.01	0.913
**BMI status**			
No excess weight	Reference	—	—
Excess weight	17.53 (14.80, 20.26)	0.87	<0.001

Data are unstandardized beta coefficients (B) with 95% confidence intervals (CI), standardized betas (β), and p-values from multivariable robust linear models (MM-estimation; lmrob, KS2014 setting) including all a priori covariates simultaneously (complete-case analytic sample, n = 747). p-values are reported to three decimal places; values <0.001 are shown as <0.001. BMI, body mass index; SES, socioeconomic status.

Table S4. Comparison of participants included in the complete-case multivariable analyses (n = 747) versus those excluded due to missing data on BIDA outcomes and/or covariates (EHDLA study).

Variable	Included (n = 747)	Excluded	p
**Age (years)**			<0.001
Median (IQR)	14.0 (2.0)	15.0 (4.0)	
**Age group**			<0.001
12–14 years old	486 (65.1%)	265 (42.0%)	
15–17 years old	261 (34.9%)	366 (58.0%)	
**Sex**			0.002
Male	340 (45.5%)	340 (53.9%)	
Female	407 (54.5%)	291 (46.1%)	
**SES**			0.943
Low	162 (21.7%)	81 (22.3%)	
Medium	396 (53.0%)	189 (51.9%)	
High	189 (25.3%)	94 (25.8%)	
**Immigrant status**			0.515
Native	567 (75.9%)	269 (73.9%)	
Immigrant	180 (24.1%)	95 (26.1%)	
**Race/ethnicity**			0.287
Caucasian	628 (84.1%)	296 (81.3%)	
Non-Caucasian	119 (15.9%)	68 (18.7%)	
**Type of schooling**			0.250
Public	600 (80.3%)	523 (82.9%)	
Private with public funds	147 (19.7%)	108 (17.1%)	
**Area of residence**			0.113
Urban	542 (72.6%)	281 (77.2%)	
Rural	205 (27.4%)	83 (22.8%)	
**Type of family**			<0.001
Nuclear	631 (84.5%)	271 (74.5%)	
Non-nuclear	116 (15.5%)	93 (25.5%)	
**Parental education (mother)**			0.099
Non-university education	535 (71.6%)	265 (76.6%)	
University education	212 (28.4%)	81 (23.4%)	
**Parental education (father)**			0.177
Non-university education	577 (77.2%)	251 (81.2%)	
University education	170 (22.8%)	58 (18.8%)	
**BMI (kg/m^2)**			0.785
Median (IQR)	21.7 (6.0)	21.8 (6.1)	
**BMI status**			0.714
No excess weight	556 (74.4%)	377 (73.3%)	
Excess weight	191 (25.6%)	137 (26.7%)	
**Number of siblings**			0.010
None	78 (10.4%)	44 (12.1%)	
One	417 (55.8%)	168 (46.2%)	
Two or more	252 (33.7%)	152 (41.8%)	
**Number of people at home**			0.013
Two or less	117 (15.7%)	69 (19.0%)	
Three	546 (73.1%)	236 (64.8%)	
Four or more	84 (11.2%)	59 (16.2%)	

Data are n (%) for categorical variables and median (IQR) for continuous variables. p-values are from chi-square or Fisher's exact tests (categorical) and Wilcoxon rank-sum tests (continuous). Percentages for categorical variables are column percentages among participants with non-missing values for each characteristic. Excluded n varies by variable because of differential missingness.
